# Developing a Conceptual Model for the Post-COVID-19 Pandemic Changing Tourism Risk Perception

**DOI:** 10.3390/ijerph18189824

**Published:** 2021-09-17

**Authors:** Chung-Shing Chan

**Affiliations:** Department of Geography and Resource Management, The Chinese University of Hong Kong, Hong Kong, China; ccs_johnson@cuhk.edu.hk

**Keywords:** changing travel behaviour, post-COVID-19 pandemic recovery, tourism disaster, tourism risk perception

## Abstract

The global coronavirus (COVID-19) pandemic has tremendously reshaped the tourism industry and destinations worldwide. Tourism destinations and the travel market require empirical research to support their post-pandemic strategies, especially in relation to the influences of changing perceptions of tourism risks, experience, and behavioural intention. This paper aims to propose a conceptual model and its hypotheses of the perceived tourism risks of natural and man-made disasters to explain the associations between the expected travel experience and ultimate travel behaviour. This paper provides a foundation for further empirical study based on a literature review and discussion. Several areas of theoretical development are identified for immediate research: (1) comparison of self-interpretation and understanding of multi-dimensional tourism risks of natural and man-made complexity in epidemics across a great variety of geographical and geo-political territories; (2) a complex web of influence to changing safety concerns and risk perception by information dissemination; (3) the effect of destination selection or hesitation in travel intention caused by changing destination image; and (4) local perception of the epidemic and health crisis. Destination authorities are recommended to (1) strengthen the preparedness and emergency responses of an effective disaster management process, (2) maintain the emotional solidarity of both tourists and local residents, and (3) mitigate multiple dimensions of the perceived risks, mainly associated with the health and psychological risks of those affected.

## 1. Introduction

Risk refers to the assessment of possibilities in which certain (negative) events may occur [1]. An individual’s perceived risk is their awareness and assessment of uncertainty and negative outcomes [2]. The global risk of natural and man-made disasters is intensifying and becoming more uncertain due to increasingly intertwined phenomena such as advancements in information and communication technology and climate change and its resultant crises. In the context of impacts on tourism and sustainability, many destinations have considered tourism as having a dual and reciprocal role as a powerhouse of economic regeneration, but it is also a vulnerable set of resources that complicates emergency planning and management for the protection of lives, properties, and environments [3,4,5,6]. Multi-dimensional globalization and rapid tourism development also expose tourists and local people to greater risks of disasters [7].

Tourism is one of the key industries that play a role in shaping and potentially reducing disaster risks [8]. Tourism risk can be induced by either a natural or a man-made disaster [9,10], although its consequential influences appear to differ based on the type of disaster and destination characteristics [11]. Tourism-related risk perception is a multi-dimensional construct [12] that extends beyond threat stimuli and temporal effects [13]. Sometimes, man-made tourism risks will increase the strength of natural disasters, eventually resulting in a greater impact on the tourism industry and destination environment [14].

Successful disaster management processes, including risk identification and disaster preparedness, have a clear connection with sustainable tourism development [14,15,16]. In the research scope of tourism, travel risk perception from an individual’s perspective is a subjective assessment of the likelihood of negative consequences of an event or a choice in the travel planning process [17,18]; this has proven to be a stronger determinant of destination selection in travel decision-making than actual existing risks [19,20]. The presence of and changes in tourism risks are regarded as potential factors affecting tourists’ collective perception of travel experience [21,22,23,24,25] and behavioural intention related to their post-disaster travel decision-making process and its related responses [26]. 

Based on the discussion of the interconnection between risk perception, perceived travel experience, and changing travel behavioural intention, this paper aims to prepare a conceptual model for further empirical investigation. Several observations on the theoretical development and policy implication are put forward. The outcome of this conceptual elucidation responds to the possible changes in the tourism industry and destinations impacted by the recent global COVID-19 pandemic. After this introductory section, the paper presents of literature review on risk perception and disaster management, specifically focusing on epidemics as a source of disasters and risks in tourism. The resultant model is proposed for hypothesis development. Lastly, theoretical, policy, and practical implications are provided for proposed hypothesis-based research, destination planning, and post-pandemic management.

## 2. Materials and Methods

### 2.1. Risk Perception and Disaster Management in Tourism

Regarding the study of tourism risks and the resultant destination management, tourism researchers have focused on specific knowledge areas with respect to different aspects of destination–disaster connection, such as risk assessment for industries and businesses [27,28], risk reduction through emergency management and public–private collaboration [3,29], impact assessment and mitigation [30,31,32], and re-visit of an affected destination [13]. 

Williams and Baláž [26], for example, examined how uncertainty and risk awareness in terms of perceived danger, objects, incidents, or activities could determine tourists’ travel decisions and behaviour. The magnitude and validity of those mediating paths and decision-making processes may be differently affected by the ways in which potential travellers perceive tourism risks, especially in the post-pandemic time, which is full of uncertainties [33]. An evaluation of perceived tourism risks toward natural and man-made disasters may display different pathways that significantly influence tourists’ perceived experience and ultimate product selection and travel decisions, although most studies report a negative relationship between perceived risk and travel intention during sudden natural and man-made disastrous incidents [7,34,35]. 

Many scholars have focused on destination- and tourism industry-oriented studies and proposed ways in which local economies can recover from disasters [36,37]; post-disaster studies in particular have sought to understand risk perception and strategies for risk reduction [38,39,40,41,42,43,44]. Based on physical characteristics, historical backgrounds, and contemporary circumstances, natural and man-made disasters carry different attributes that might eventually lead to different risk perceptions and changes each time they occur. Some researchers have examined the risks caused by natural and man-made disasters to tourist destinations (e.g., [15,45,46,47,48]). However, a knowledge deficiency exists, particularly for those circumstances after the occurrence of large-scale regional or even global hazards and disasters [16]. Particularly in the recent coronavirus disease (COVID-19) pandemic, similar global crises to all industries may become more frequent and must be tackled across different areas of economic activity [49,50,51]. Li et al. [52] highlighted the dearth of studies focused on the potential personal impacts from disease outbreak and the subsequent pandemic, and therefore applied construal level theory to develop a theoretical foundation for further research based on the relationship between psychological distance, perceived risk, and tourism crises.

### 2.2. Epidemics as a Source of Disasters and Risks in Tourism

The outbreak of diseases such as avian flu, severe acute respiratory syndrome (SARS), Middle East respiratory syndrome (MERS), and the recent COVID-19 and their epidemics have imposed tremendous economic impacts on the global tourism industry and socio-political situations in many developed and developing countries [53]. 

The tourism risk perception of epidemics has been intensified due to the growth in international tourist flows and movement of a large number of travellers [11,33,54]. This problem is relevant to gaps in socio-economic characteristics, sanitation standards, and cultural differences between countries [55]. Having some public health policy controversies [56], these cases of epidemics brought about reductions in tourist arrivals and market demand, as well as changes in tourist behaviour and destination selection [20,54,57,58,59,60]. 

A real-world example was the reduction in employment of about 3 million people during the SARS outbreak in Asian countries or territories, including China, Hong Kong, Vietnam, and Singapore, in addition to a revenue loss of over USD 20 billion [61]. With gradual and effective cross-border restrictions and other health measures, Hong Kong, mainland China, and other countries in Asia have started to recover their economic activities and people’s movement. Therefore, timely research both globally and regionally is essential to respond to the call for both testing a conceptual model of socio-environmental and psychological disciplines through empirical studies [7,52,57,62,63], and comparing differences in risk perception among tourist segments for policy implications and practical recommendations [41,52]. Such research directions will help to understand the reactions of potential travellers for a more sustainable and efficient recovery.

Confidence recovery through safety and security enhancement is one of the UNWTO’s urgent actions [64]. Researchers have recognized tourism risks induced by both natural and man-made disasters as a critical factor affecting tourist behavioural intention collectively (e.g., [48,58,63,65]). However, tourism as a field of research is still only a small component of disaster-related research from the past few decades [7,39,66]. Several areas of knowledge deficiency can be observed.

First, tourism researchers have been providing more updated conceptual and empirical studies on tourist, industry, and destination responses to the impacts of COVID-19 (e.g., [50,52,67,68,69,70,71,72]). Compared with other epidemics, the COVID-19 pandemic has evoked panic among people worldwide, which leads to a proximate temporal distance to disease outbreak, close spatial distance, and strong hypotheticality of transmission and serious outcomes [52]. 

Second, many previous studies on tourism risk perception have described and explained specific disasters and incidents [9,10,21,23,24,25,46,52,63,68,73,74]. Li et al. [52] have proposed six categories of risk perception attributes, including health risk, psychological risk, social risk, performance risk, image risk, and time risk, which are relevant to both natural and man-made conditions, indicating varied temporal, spatial, and social distances. Risk perception is often derived from and exaggerated by emotional and psychological factors rather than being solely based on facts [69,72]. However, the existing literature has yet to develop a mature theoretical base to holistically model the risk-associated attributes to cover a wider range of natural and man-made disasters such as epidemics [75,76], and to test the subsequent empirical cases and practical implications to various destinations. It is therefore essential to broaden and deepen the understanding of this risk-experience–behavioural relationship in tourism literature to draw important insights into how tourism risks may influence tourists’ travel decisions, behavioural intentions, and destination image.

Third, previous studies have generally agreed that risk perception does influence destination selection [7,10,19,20,35,40,41,68,77,78], although the influence tends to be dynamic and occasionally uncertain due to more frequent incidences of hazards and crises today. Some researchers, however, discovered that the level of perceived risk only led to a substitution of destinations rather than a permanent termination of all international travel plans [11,79]. When leisure constraint theory was applied to the context of tourism risks and uncertainty, it was observed that risk perception could only partially affect the whole process of travel intention and behaviour, thus strengthening the influence of other factors such as interpersonal and structural constraints in determining the final travel decision [80].

### 2.3. Conceptualizing COVID-19 Pandemic Changing Tourism Risks, Travel-Related Experience, and Behaviour

The World Health Organization (WHO) has estimated that billions of people are at a public health risk [81], which also extends to different risks for the travel and tourism industries [81]. From the macro-economic perspective, Rosselló et al. [54] analyzed the economic benefits of the eradication of Dengue, Ebola, Malaria, and Yellow Fever for the tourism sector, and further proposed that the economic benefits of health policies should be quantitatively included in future international health assessment programmes and development plans. Disease eradication should also be evaluated beyond GDP growth and extended to potential multiplicative effects on tourism attractiveness, destination image, inbound and outbound tourist experience, and behaviour [34,48,57,82,83,84]. By observing the recent decades of global health and epidemic issues, it can be predicted that similar global epidemics and crises are likely to become more frequent, impactful, unpredictable, and difficult to manage and resolve in all economic sectors worldwide. 

The recent COVID-19 pandemic is a global catastrophe that has drastically hindered the operation of tourism industries, destinations, and countries [49,51]. Across all major economic sectors, tourism has been the worst affected by this pandemic. Relatedly, the socio-economic sustainability of many countries as tourist destinations is also facing a collapsing situation. The United Nations World Tourism Organization (UNWTO) has already articulated strategies by “standing in solidarity with affected countries” and “emphasizing tourism’s proven resilience and by standing ready to support recovery” [85]. The effectiveness of these two actions is largely determined by the provision of up-to-date and reliable information from different sources through academic and policy-supportive research.

In connection with individual tourists, the perceived risk of an epidemic affects tourist behaviour, especially in their choice of tourist destination [20,54,78], mediated by uncertainty and concerns surrounding public health [59] and transformed destination image [7,84]. There is an urgent need for greater crisis preparedness and disaster management strategies; thus, it is essential to understand aspects of the tourist perception of disaster (including a combined nature of natural and man-made disasters), such as health risks, experiences, and changing travel behaviour [43,69], particularly due to hesitation to restart a journey after an epidemic [86].

## 3. Results and Discussion—Proposed Model Development

### 3.1. Structuring the Relationship between Constructs

Tourism risks are categorized into risks related to natural disasters and man-made disasters. Infectious disease outbreak can be regarded as a form of natural disaster (e.g., biological issue and virus mutation) and man-made disaster (e.g., new travel or transport-related inflections, environmental contamination, success or failure of disease control measures) [48,87], although some scholars believe that epidemics indicate human-borne risk [69]. The connection between the two constructs (risk perception and travel decision-making) may be mediated by the attributes of perceived or expected travel experience [21,22,23,24,25]. This mediation effect can be functioning between the two constructs and can be verified in the postulated model. 

One of the most relevant studies associated with the proposed conceptual framework is provided by Wong and Yeh [86], who confirmed that tourist risk perception positively influences travel decision, but tourist knowledge can moderate this relationship. The model can extend further with three changes: (1) consideration of perceived risks induced by both natural and man-made disasters, (2) replacement of the knowledge dimension by expected or perceived travel experience due to the pandemic, and (3) detection of some changes in travel behaviour. 

### 3.2. Hypotheses Development

Based on the conceptual model, a series of directional relations can be established and hypothesized for empirical testing. Tourism risks are complex crises in the tourism industry that take many shapes and forms [9]. Tourism risks associated with natural or man-made disasters are inter-connected [10], but the risk perception attributes are multi-dimensional in an epidemic or a health incident [52]. Man-made actions often increase the possibility and impact of natural disasters [65,76], and sometimes may reduce the impact of natural disasters [88,89]. This nature–human connection is reinforced by the perception of sustainable management and environmentally responsible behaviour [34], as well as the socio-cultural backgrounds of people [15]. The first hypothesis is therefore denoted by the following:

**Hypothesis** **1** **(H1).** *Risk perception of man-made disasters is positively related to that of natural disasters*.

A natural disaster refers to any natural event, which could be sudden or progressive, causing hazardous impacts on the affected area in which the local communities must take exceptional measures in response to cope with the event [8,88]. Compared to man-made events, natural hazards are much more difficult to manage, if not totally unpredictable [7,9,90]. The overall perceived risk of both natural and man-made disasters could be manifested in tourists’ expected travel experience and behaviour through the influence of the history of hazards in a geographical territory [15,21,22,23,24,48,73,87,91]. Risk perception goes beyond actual experience and threat stimuli to subjective expectation or uncertainty [92]. Although these two kinds of tourism risk appear to be correlated, their associated behaviours tend to have distinctive effects on perceived travel experience and behaviour [34,48,93]. As a result, three hypotheses are developed:

**Hypothesis** **2** **(H2).** *Risk perception of man-made disasters positively determines the perceived travel experience*.

**Hypothesis** **3** **(H3).** *Risk perception of natural disasters positively determines the perceived travel experience.*

**Hypothesis** **4** **(H4).** *Perception of expected travel experience is positively related to travel behavioural intention.*

Scholars have suggested that changing travel experience is a mediator in the tourism risk–behaviour relationship [10,20,21,24,25,45,46]. Tourists also tend to reduce travel risk and avoid a possible trip based on their individual feelings [7,46,77] and their willingness to visit, re-visit, or not visit a destination [13,73]. However, scholars have also critically proposed that the subjective side of risk perception should be properly addressed in further study [13,25,92]. Consequently, it is also essential to verify whether the perceived risks of man-made and natural disasters directly influence tourists’ travel behavioural intention. Therefore, another two hypotheses are proposed:

**Hypothesis** **5a** **(H5a).** *Risk perception of ma* *n-made disasters and travel behavioural intention mutually influence each other.*

**Hypothesis** **5b** **(H5b).** *Risk perception of natural disasters and travel behavioural intention mutually influence each other.*

**Figure 1:**
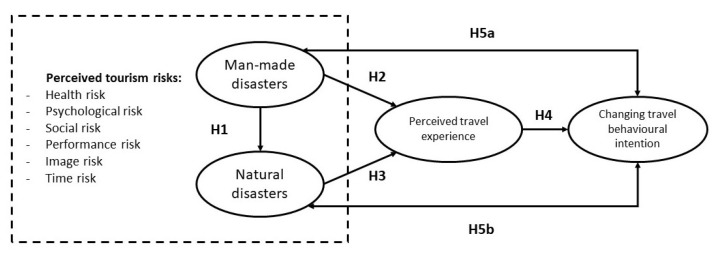
Conceptual model and hypotheses of the relationship between perceived tourism risks, perceived travel experience, and changing behavioural intention.

Based on the hypotheses illustrated above, in this study, the conceptual framework shown in Figure 1 has been proposed. All the above hypotheses postulate a conceptual model that requires verification by empirical findings and data from real-world environments.

## 4. Conclusions and Implications

Based on the postulation of the relationship between risk perception, perceived travel experience, and the changing travel behavioural intention, the overall impact of the COVID-19 pandemic on particular destinations can be detected and verified through empirical studies on the tourists of both generating locations and destinations. Whereas the inbound market of a destination indicates the potential change in the overall tourism demand for that destination, its outbound market represents the possible opportunities for proximity tourism development and preferred themes of attractions and products [70].

### 4.1. Theoretical Implications and Further Research

The proposed conceptual model postulates the intermediation of potential tourists’ risk perceptions of natural and man-made disasters and their possible travel behavioural changes. This model is consistent with the critical review on a series of research agenda addressing the current COVID-19 pandemic impacts [87]. This relationship provides several theoretical insights and practical implications.

First, the perception of possible tourism risk and safety may directly induce subsequent travel decision-making and behaviour, or the hesitation to these decisions [7,10,77,94,95,96]. The self-interpretation and understanding of tourism risk may vary between individual tourists, especially during the occurrence of a disaster or a combination of disasters [43,86]. Whereas destinations are more likely to encounter natural and man-made complexity during a hardly defined and managed incidence such as an epidemic [75,76], the impact of the pandemic may be widespread across a great variety of geographical and geo-political territories. The tourism risk perception is therefore more than merely a single indication of reported value, but is rather a series of risk dimensions, which may be better conceptualized by the application of psychological distance and construal level theory for further study [52,97]. 

Second, information dissemination and exchange across various channels have a complex influence on changing safety concerns and risk perceptions, which may eventually mitigate anxiety and foster tourism recovery given positive and accurate sources of information [98,99]. Social media tend to easily disseminate both positive and negative messages and sometimes exacerbate image issues to their users [12,84,100]. Considering the duration of temporal scale and proximity in the spatial extent of the COVID-19 pandemic, the epidemic and the subsequent recovery stages all display a high degree of uncertainty similar to but more severe than that of previous epidemics [59,92]. As such, much of the information may change over time, which causes further psychological distance and less detail-oriented and spontaneous trait inferences [97], and sometimes results in irrational or unethical risk perceptions and attitudes [101]. As a result, further research must be conducted to understand how post-disaster information is transmitted among stakeholders and potential tourists, and how the accuracy and effectiveness of that information can be used and managed for tourism recovery [16].

Third, a possible change in destination image is regarded as an attribute of expected travel experience that may eventually influence destination selection or hesitation in travel intention [16,34,73,78,102,103,104]. During the pandemic period, destination image is dynamic in terms of geographical extent, in which the changing epidemic transmission and influence across territories may be different between countries according to the infection and recovery statistics, and between cities due to specific lockdown and social distancing policies. According to Li et al. [52], image risk as one of the perceived risk dimensions may emerge to determine destination choice and evaluation [7,84,104], which is often triggered by media coverage [67,83,105]. In this COVID-19 pandemic, an essential area of investigation is the divergent effects of anti-pandemic policy implementation, projected image by governments, and communicated image by media of different territories on the changing perceived destination image of potential tourists, which is not comparable by the scale and impacts of all previous cases of epidemics [83,87,105,106,107].

Furthermore, the application of social exchange theory to local cost–benefit evaluation and intention to respond and act has been well documented in tourism research [108,109]. However, this area of theoretical development of local perception in epidemic and health crises is still under-researched [110]. According to Joo et al. [69], there is an apparent need to deepen the theoretical understanding of how perceived risk affects perceived benefits and costs, and ultimately shapes the actions for tourism (re)development. Local residents and communities are highly relevant to risk exposure and important in the reshaping of destinations after the COVID-19 pandemic [51,87,111]. The conceptual model proposed in this paper lays down a foundation for future research on different actors in tourism, which is applicable to different destinations or risks. Further research is required to test the robustness of the conceptual model, especially its applicability to different forms of tourism [52] and across stages of the disaster management process [16].

### 4.2. Policy and Practical Implications

Tourism is a chain of stakeholders, resources, and activities involving various interconnected industries and destination environments [112]. The COVID-19 pandemic heavily impacts all these operations with different levels of suspension, economic loss, and crisis. Tourism stakeholders, including different levels of government, national and local tourism authorities, destination management and marketing organizations, tourism industries and businesses, and local communities and individual tourists, are all relevant, to varying degrees, to the process of disaster risk reduction and impact minimization and mitigation [3]. 

Regarding the disaster management process, the global COVID-19 pandemic represents an apparent breakdown of public health systems and disaster management systems in many countries [113]. More policies and practices toward providing assistance to international tourists in emergency situations, such as a rapid disease outbreak and retreat from destinations, must be effectively implemented through territorial and sectoral collaboration [114]. Such suspension of travel and societal activities is unavoidable, but the need for a rapid outbound departure of tourists and the danger of disease transmission through transport are paradoxical. During normal periods, tourism destinations must strengthen their capacity for monitoring, assessing, and reacting to disasters effectively. Destinations may consider imposing a tourism tax on both tourists and tourism businesses. The accumulated taxes can be transformed into a disaster management fund which can be further established and invested in contingency financial support for the most vulnerable groups in the tourism industry during disaster occurrence and post-disaster recovery [49,51]. 

The post-pandemic risk perception of individuals is determined by uncertainty in disease-related information [59,92]. It is extremely important to mitigate the perceived risks of both tourists and local residents, and to maintain their emotional solidarity through efficient policy responses to public concerns. The key to achieving this is the provision and dissemination of accurate information about destination safety [98] and, in the case of epidemics, guidance for departures, precautions, infections, and medication for tourists [24,52]. The overall national information about the epidemic statistics must be centralized, whereas de-centralized administration for emergency arrangements is more effective than a top-down governance because epidemic transmission and conditions can be very diverse across localities [49]. The mitigation of perceived risks allows residents to feel safer and less threatened by tourists and other outsiders [69,111].

According to Li et al. [52], health risks and psychological risks are the fundamental starting points for restoring the confidence of potential tourists to travel. Health risks influence the well-being and travel behavioural intention of a number of stakeholders of the tourism and local economies of the destination [24,46,57,115]. More than medication and visible precautions, the psychological risks should also be addressed by the public authorities to mitigate public fear, anxiety, and mental discomfort [59,116,117]. Behavioural changes may be revealed in specific areas of travel, such as destination or activity selection [118,119] and the choice of transportation mode [120,121].

The COVID-19 pandemic is certainly the most tremendous disastrous event in the past few decades. The pandemic and its resultant travel suspensions and large-scale socio-economic crises have affected the tourism industry and destinations worldwide. This pandemic underlines that tourism must be re-defined, transformed, and understood in the greater global socio-economic and political context. The postulated relationship between risk perception, perceived travel experience, and the changing travel behavioural intention connects with the opportunity for a paradigm shift from consumptive to sustainable and regenerative tourism [70,122,123]. Greater attention must be paid to ecological, ethical, and health issues rather than conventional economic and capitalist practices.

## Data Availability

No new data were created or analyzed in this study. Data sharing is not applicable to this article.
